# Arsenic exposure and respiratory outcomes during childhood in the INMA study

**DOI:** 10.1371/journal.pone.0274215

**Published:** 2022-09-09

**Authors:** Antonio J. Signes-Pastor, Susana Díaz-Coto, Pablo Martinez-Camblor, Manus Carey, Raquel Soler-Blasco, Miguel García-Villarino, Ana Fernández-Somoano, Jordi Julvez, Paula Carrasco, Aitana Lertxundi, Loreto Santa Marina, Maribel Casas, Andrew A. Meharg, Margaret R. Karagas, Jesús Vioque-Lopez

**Affiliations:** 1 Unidad de Epidemiología de la Nutrición, Universidad Miguel Hernández, Alicante, Spain; 2 CIBER de Epidemiología y Salud Pública (CIBERESP), Instituto de Salud Carlos III (ISCIII), Madrid, Spain; 3 Instituto de Investigación Sanitaria y Biomédica de Alicante (ISABIAL), Alicante, Spain; 4 Department of Epidemiology, Geisel School of Medicine, Dartmouth College, Lebanon, NH, United States of America; 5 Biomedical Data Science Department, Geisel School of Medicine, Dartmouth College, Lebanon, NH, United States of America; 6 Institute for Global Food Security, School of Biological Sciences Building, Queen’s University Belfast, Belfast, Northern Ireland, United Kingdom; 7 Epidemiology and Environmental Health Joint Research Unit, FISABIO−Universitat Jaume I−Universitat de València, Valencia, Spain; 8 Unit of Molecular Cancer Epidemiology, University Institute of Oncology of the Principality of Asturias (IUOPA)–Department of Medicine, University of Oviedo, Oviedo, Asturias, Spain; 9 Institute of Health Research of the Principality of Asturias (ISPA), Oviedo, Spain; 10 Institut d’Investigació Sanitària Pere Virgili, Hospital Universitari Sant Joan de Reus, Reus, Spain; 11 ISGlobal- Instituto de Salud Global de Barcelona-Campus MAR, Parc de Recerca Biomèdica de Barcelona (PRBB), Barcelona, Spain; 12 Department of Medicine, Universitat Jaume I, Castellón de la Plana, Spain; 13 Department of Preventive Medicine and Public Health, UPV/EHU, Leioa, Basque Country, Spain; 14 Health Research Instititue, Biodonostia, Donostia-San Sebastian, Spain; 15 Department of Health of the Basque Government, Public Health Division of Gipuzkoa, Donostia-San Sebastián, Spain; 16 Universitat Pompeu Fabra, Barcelona, Spain; University of Rajshahi, BANGLADESH

## Abstract

Ingested inorganic arsenic (iAs) is a human carcinogen that is also linked to other adverse health effects, such as respiratory outcomes. Yet, among populations consuming low-arsenic drinking water, the impact of iAs exposure on childhood respiratory health is still uncertain. For a Spanish child study cohort (INfancia y Medio Ambiente—INMA), low-arsenic drinking water is usually available and ingestion of iAs from food is considered the major source of exposure. Here, we explored the association between iAs exposure and children’s respiratory outcomes assessed at 4 and 7 years of age (*n* = 400). The summation of 4-year-old children’s urinary iAs, monomethylarsonic acid (MMA), and dimethylarsinic acid (DMA) was used as a biomarker of iAs exposure (∑As) (median of 4.92 μg/L). Children’s occurrence of asthma, eczema, sneeze, wheeze, and medication for asthma and wheeze at each assessment time point (i.e., 4- and 7-year) was assessed with maternal interviewer-led questionnaires. Crude and adjusted Poisson regression models using Generalized Estimating Equation (GEE) were performed to account for the association between natural logarithm transformed (ln) urinary ∑As in μg/L at 4 years and repeated assessments of respiratory symptoms at 4 and 7 years of age. The covariates included in the models were child sex, maternal smoking status, maternal level of education, sub-cohort, and children’s consumption of vegetables, fruits, and fish/seafood. The GEE—splines function using Poisson regression showed an increased trend of the overall expected counts of respiratory symptoms with high urinary ∑As. The adjusted expected counts (95% confidence intervals) at ln-transformed urinary ∑As 1.57 (average concentration) and 4.00 (99^th^ percentile concentration) were 0.63 (0.36, 1.10) and 1.33 (0.61, 2.89), respectively. These exploratory findings suggest that even relatively low-iAs exposure levels, relevant to the Spanish and other populations, may relate to an increased number of respiratory symptoms during childhood.

## 1. Introduction

The International Agency for Research on Cancer has classified ingested inorganic arsenic (iAs) as class 1 human carcinogen [1]. Exposure to iAs also relates to other non-carcinogenic health outcomes among highly exposed populations [2–6]. There is also a growing body of evidence suggesting that even low levels of iAs chronic exposure relate to deleterious health impacts [7–10]. Low-level chronic exposures refer to populations with access to drinking water that complies with the EU, US and WHO maximum arsenic level of <10 μg/L, such as the Spanish population and others [11–13]. In such populations, food intake is considered the major source of exposure to iAs [14–17].

High-level exposure to iAs such as that from consumption of contaminated water is associated with bronchiectasis, chronic obstructive pulmonary disease, chronic bronchitis, and decreased lung function [10, 18–26]. Inorganic arsenic crosses the placenta, and even relatively low exposure levels during gestation related to an increased risk of infections during the first year of life of children from the New Hampshire Birth Cohort study (NHBCS) [9, 27, 28]. A recent study from the same cohort reported that maternal exposure during pregnancy were also associated with a reduced lung function of 7-year-old children [29]. These findings and others support that even low-level iAs exposure *in utero* and early life relates to detrimental health effects that may last throughout the lifespan [28–30]. Infants and young children are particularly vulnerable to the toxic effects of iAs, and often have higher ingestion of iAs compared to adults [15, 27, 31].

The iAs is metabolized in the liver through a series of reduction and oxidative methylation reactions that ends with the excretion of monomethylarsonic acid (MMA), dimethylarsinic acid (DMA), and unmetabolized iAs in urine within a few days. The iAs methylation process is considered a mechanism for detoxification; however, it generates intermediate trivalent metabolites (i.e., monomethylarsonous acid and dimethylarsinous acid) that are shown to be more toxic than their parent compounds and are involved in the mechanism of arsenic toxicity [32–35]. Thus, the summation of urinary iAs, MMA and DMA (∑As) is commonly use as iAs exposure biomarker in environmental epidemiology studies [24, 36–40]. Ingestion of organosenical compounds such as arsenobetaine (AsB) are excreted in the urine unchanged and do not appear to pose toxicity [37, 38].

Rice and rice-based products may contain high iAs concentrations that relate with an increased iAs metabolites content in urine [41–43]. Among NHBCS infants, introduction of rice cereals was associated with increased susceptibility to respiratory infections and allergy during their first year of life [44]. Maximum levels of iAs in rice and rice-based products have been advised or regulated to reduce exposure [45–47]. The most restrictive level of 100 μg/kg of iAs has been stablished for rice destined to produce food for infants and young children [46]. However, a safe level of exposure to iAs has not been observed yet and the health effects of exposure during childhood required further investigations [1, 48, 49].

In this study, we aimed to explore the association between iAs exposure, evaluated using urinary ∑As concentrations, in 4-year-old children from the Environment and Childhood project (“INMA—INfancia y Medio Ambiente”) in Spain and respiratory symptoms evaluated at 4 and 7 years of age using face-to-face interviewer-led questionnaires.

## 2. Methods

### 2.1. Study population

The study population here came from the INMA project, a prospective population-based birth cohort study conducted in several areas of Spain [50, 51]. Briefly, INMA recruited women at the beginning of their pregnancy (2003–2008) and they were followed up until delivery (*n* = 2625), then their children were enrolled and followed-up. In this study, we selected a random subset of children evenly distributed by cohort including Asturias (*n* = 100), Guipuzkoa (*n* = 100), Sabadell (*n* = 100), and Valencia (*n* = 100) [36]. The final dataset for statistical analysis contained 339 participants without missing values in urinary arsenic species concentrations, cumulative respiratory symptoms at each time point, and the confounding variables (i.e., case-complete approach). Informed consent was obtained from all INMA participants before each phase, and the hospital ethics committees in all participating regions approved the study (i.e, Central University Hospital of Asturias, Donostia Hospital, Health Consortium of Catalonia—Medical Assistance Municipal Institute, and University Hospital “La Fe”).

### 2.2. Laboratory analysis

Spot urine samples were collected in 100 mL polyethylene containers during the pediatric follow-up review at 4 years and stored at -80°C. One aliquot of the sample from each of the participants was sent to the Institute of Global Food Security, Queen’s University Belfast, Northern Ireland to be analyzed for arsenic speciation. To carry out the urinary arsenic speciation analysis, the urine samples were first centrifuged with a Sorvall Legend RT at 4500 g. Then, a 1 ml aliquot was transferred to a 2-ml polypropylene vial with 10 μl of analytical grade hydrogen peroxide to convert any arsenite to arsenate to facilitate subsequent chromatographic detection. The arsenic speciation analysis included iAs (arsenite + arsenate), MMA, DMA, and AsB using ion chromatography (IC)-inductively coupled plasma mass spectrometry (ICPMS) [8, 36, 42]. The urine samples were analysed in different batches, including blanks and replicate samples of the certified reference material (CRM) ClinChek—Control level I. Based on *n* = 33 urine CRM ClinChek—Control level I samples, the urinary arsenic species recovery (mean ± standard error) was 115 ± 2% for iAs, 97 ± 2% for MMA, 94 ± 2% for DMA, and 90 ± 2% for AsB. The LOD for arsenic speciation was 0.011 μg/L [36]. The gravimetric summation of the urinary arsenic species in μg/L (∑As = iAs + MMA + DMA) was calculated as an estimate of iAs exposure. The ∑As accounts for the individual fractions of iAs and the metabolites, excluding the non-toxic AsB found in fish/seafood [24, 36–40]. Urine samples were adjusted for urine dilution using specific gravity measured with a clinical refractometer [36].

### 2.3. Respiratory outcomes

The primary outcomes of this study were respiratory-related symptoms. Interviewer-led questionnaires given to the mothers collected information on the occurrence (yes/no) of asthma, eczema, sneeze, wheeze, and medication for asthma and wheeze at ages of 4 and 7 years. The questionnaire was the Spanish, Catalan or Basque version of the validated International Study of Asthma and Allergies in Childhood (ISAAC) questionnaire, depending on the primary language of the mother [52, 53].

The occurrence of 1. wheeze, 2. wheeze medication, 3. asthma, 4. asthma medication, 5. eczema, or 6. sneeze was defined as a positive answer to the following questions i) at 4 years of age, 1.”Has your child ever had wheezing in the chest in the past 12 months?“, 2.”Has your child taken any medication for wheezing in the past 12 months?“, 3.”In the last 12 months, has your child had ever suffered asthma?“, 4.”Has your child had medical assistance due asthma?“, 5.”In the last 12 months, has your child had ever suffered atopic eczema?“, and 6.”In the last 12 months, has your child had ever suffered allergic rhinitis?“, and ii) at 7 years of age, 1.”Has your child had wheezing or whistling in the chest in the past 12 months?“, 2.”Has your child taken any medication for wheezing in the last 12 months?“, 3.”Has your child ever been diagnosed by a doctor as having asthma?“, 4.”Any medicines for asthma/breathing difficulties in last 12 months?“, 5.”Ever diagnosed by doctor with having eczema/atopic dermatitis?“, and 6.”In the last 12 months, has your child had problems with sneezing, runny, blocked nose when not cold or flu?“.

### 2.4. Covariates

A maternal questionnaire was administered during the 1^st^ and 3^rd^ trimester of pregnancy to gather information regarding parental sociodemographic and socioeconomic characteristics (e.g., living area, education, and social class). Children’s diet was assessed with validated food frequency questionnaires (FFQ) asking about consumption of food items and portion sizes appropriate for children in the previous year [54, 55]. Parents or children’s guardians completed the FFQ during a personal interview with trained personnel as described elsewhere [42].

### 2.5. Statistical analyses

For the main statistical analysis, a case-complete approach was followed with a dataset of 339 participants as mentioned earlier. Crude and adjusted Poisson regression models using Generalized Estimating Equation (GEE) were performed. This approach allowed to account for the association between natural logarithm (ln) transformed urinary ∑As in μg/L at 4 years of age and repeated assessments of respiratory symptoms at 4 and 7 years. The ∑As was ln-transformed owing to its positive skewness. The covariates included in the adjusted models were child sex (boys or girls), and maternal smoking status (“Have you ever smoked?”—binary), maternal level of education (primary, secondary, or university studies), sub-cohort (Asturias, Gipuzkoa, Sabadell, or Valencia), and child consumption of vegetables (g/day), fruits (g/day) and fish/seafood (g/day) calorie adjusted using the residual method [56]. The covariates were selected based on previous studies and the Directed Acyclic Graph using the DAGitty software (**S1 Fig—Directed acyclic graph or causal Bayesian network created using DAGitty browser-based environment**) [9, 57, 58].

This study included various sensitivity analyses. First, the Poisson regression models for each assessment time point (i.e., 4- and 7-year) were carried out. The cumulative expected counts of respiratory events assessed at both time points were also modelled. Second, the study included logistic regression spline functions between ln-transformed urinary ∑As and each respiratory outcome assessed at 4 and 7 years of age combined and separately. Third, it modelled GEE—logistic regression spline functions between ln-transformed ∑As and each cumulative respiratory symptom assessed at 4 and 7 years of age. Finally, the main GEE- Poisson regression models were performed including (1) iAs + MMA and (2) DMA urinary concentrations ln-transformed as dependent variables.

A nominal level of 0.05 to define associations as statistically significant was applied. Concentrations below the limit of detection (LOD) were replaced with the ½ LOD. The ½ LOD value was used for statistical analysis and graphics generation purpose. The original data contained 10 (2.5%), 29 (7.2%), and 17 (4.2%) urinary DMA, MMA, and iAs concentrations <LOD [42]. We calculated the LOD as the mean of the blank concentrations plus three times the standard deviation of the blank concentrations multiplied by the dilution factor. The R software version 4.0 was used to conduct all statistical analyses and graphics [59]. Particularly, we used the functions *glm*, *rcs*, and *geeglm* from the package *stats*, *rms*, and *geepack*, respectively.

## 3. Results

Maternal median age of enrolment was 39 years of age, and our child study population was evenly distributed among boys and girls. Children’s respiratory symptoms were assessed at the median (first and third quartile (Q1—Q3)) of 4.42 (4.36–4.49) and 7.75 (7.49–8.03) years of age. Their median (Q1—Q3) urinary concentration was 4.92 (2.94–7.80), 0.36 (0.21–0.57), 0.43 (0.25–0.69), 4.07 (2.22–6.13), and 9.61 (2.89–35.12) μg/L for ∑As, iAs, MMA, DMA, and AsB, respectively (**Table 1**). Urinary iAs, MMA, and DMA represented the median (Q1—Q3) of 8.4% (4.9–12.1), 9.8% (5.7–12.9), and 81.4% (75.5–87.1) of ∑As concentrations in μg/L, respectively. The interclass correlation coefficient (ICC) for the concentrations of iAs, MMA, and DMA was 0.063 with a 95% confidence interval (CI) ranging from -0.001 to 0.130.

**Table 1:** Selected characteristics of study mothers and children from INMA.

Variables	Original sample (*n* = 400)
**Maternal characteristics**:	*n* = 339
Age of enrolment	39 (21; 29–34; 43)
Smoking status: no | yes	160 (40.0%) | 179 (44.8%)
Maternal education: Primary | Secondary | University	65 (16.3%) | 141 (35.3%) | 133 (33.3%)
**Children characteristics:**	*n* = 339
Sex: boys | girls	169 (42.3%) | 170 (42.5%)
Specific gravity	1.02 (1.00; 1.01–1.02; 1.03)
Urinary iAs (μg/L)	0.36 (0.01; 0.21–0.57; 10.98)
Urinary MMA (μg/L)	0.43 (0.01; 0.25–0.69; 6.08)
Urinary DMA (μg/L)	4.07 (0.01; 2.22–6.13; 81.4)
Urinary ∑As (μg/L)	4.92 (0.21; 2.94–7.80; 84.46)
Urinary AsB (μg/L)	9.61 (0.06; 2.89–35.12; 3569.48)
**At 4-year time point:**	
Age	4.42 (4.09; 4.36–4.49; 5.41)
Wheeze; no | yes	*n* = 337; 262 (65.5%) | 75 (18.8%)
Wheeze medication (4 years); no | yes	*n* = 93; 81 (20.3%) | 12 (3.0%)
Asthma; no | yes	*n* = 337; 327 (81.3%) | 10 (2.5%)
Asthma medication (4 years); no | yes	*n* = 334; 325 (81.3%) | 9 (2.3%)
Eczema; no | yes	*n* = 336; 276 (69.0%) | 60 (15.0%)
Sneeze; no | yes	*n* = 334; 327 (81.8%) | 7 (1.8%)
**At 7-year time point:**	
Age	7.75 (5.36; 7.49–8.03; 9.51)
Wheeze; no | yes	*n* = 337; 294 (73.50%) | 43 (10.75%)
Wheeze medication; no | yes	*n* = 93; 88 (22.0%) | 5 (1.3%)
Asthma; no | yes	*n* = 337; 319 (79.8%) | 18 (4.5%)
Asthma medication; no | yes	*n* = 334; 293 (73.3%) | 41 (10.3%)
Eczema; no | yes	*n* = 336; 232 (58.0%) | 104 (26.0%)
Sneeze; no | yes	*n* = 334; 254 (63.5%) | 80 (20.0%)
**At 4- and 7-year time points:**	
Wheeze; no | yes	*n* = 337; 245 (61.3%) | 92 (23.0%)
Wheeze medication; no | yes	*n* = 93; 78 (19.5%) | 15 (3.8%)
Asthma; no | yes	*n* = 337; 318 (79.5%) | 19 (4.8%)
Asthma medication; no | yes	*n* = 334; 291 (72.8%) | 43 (10.8%)
Eczema; no | yes	*n* = 336; 214 (53.5%) | 122 (30.5%)
Sneeze; no | yes	*n* = 334; 251 (62.8%) | 83 (20.8%)
Overall outcomes in each time point: 4-year | 7-year	201 | 295

Continuous values are reported as median (minimum; first and third quartile (Q1—Q3); maximum), and categorical values as relative and absolute frequencies. The final dataset for statistical analysis contained 339 participants without missing values in urinary arsenic species concentrations, cumulative respiratory symptoms at each time point, and the confounding variables. The case-complete datasets for each respiratory symptom are also shown. The percentages are calculated from the original sample (*n* = 400), and thus they might not sum 100%.

**Fig 1:**
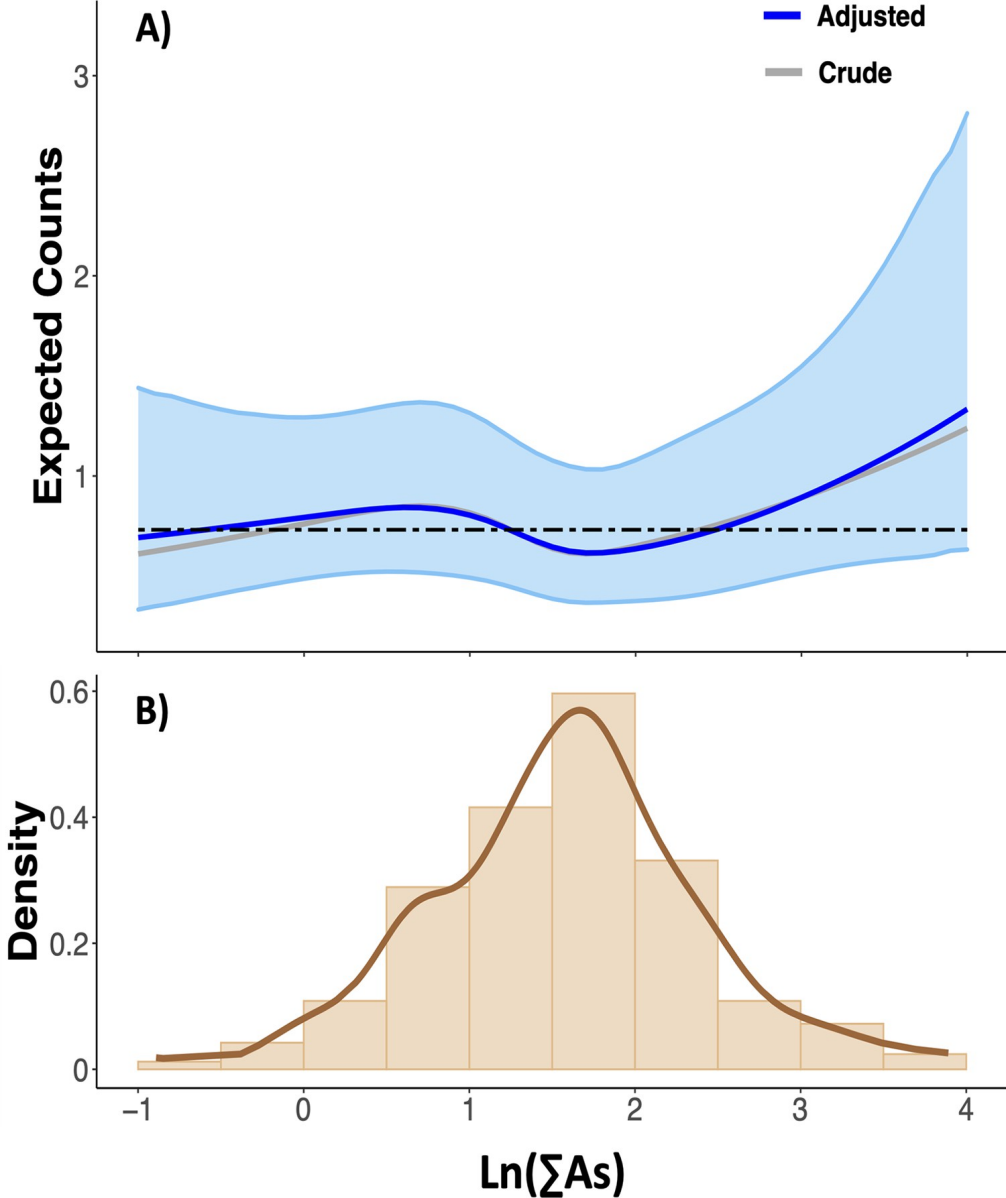
Generalized Estimating Equation (GEE)—Poisson regression spline function between natural ln-transformed urinary arsenic concentrations (∑As) and expected respiratory symptoms at 4 and 7 years of age. The ∑As is in μg/L. Case-complete approach (i.e., participants with missing values in the dependent, independent, or adjustment variables were not included in the analysis). *N* at 4 years of age equal to 339 including 0 counts = 216 (63.7%), 1 count = 73 (21.6%), and ≥2 counts = 50 (14.7%). *N* at 7 years of age equal to 339 including 0 counts = 170 (50.1%), 1 count = 101 (29.8%), and ≥2 counts = 68 (20.1%). ∑As = iAs + MMA + DMA adjusted for specific gravity. **Fig. A**) Generalized Estimating Equation (GEE) using family equal Poisson and splines functions. Respiratory symptoms include asthma, asthma medication, wheeze, wheeze medication, sneeze, and eczema. The grey line shows the crude model. The dark blue line shows the adjusted models for child sex (boys or girls), and maternal smoking status (“Have you ever smoked?”—binary) and level of education (primary, secondary, or university studies), cohort (Asturias, Gipuzkoa, Sabadell, or Valencia), and calorie adjusted consumption of vegetables (g/day), fruits (g/day) and fish/seafood (g/day) at 4 and 7 years of age. The blue shade shows the 95% confidence interval of the adjusted model. The black dashed line shows the average expected counts (0.73). To facilitate interpretation, the crude and adjusted estimates (95% confidence intervals) at ln-transformed urinary ∑As 1.57 (average concentration), and 4.00 (99^th^ percentile concentration) were 0.62 (0.48, 0.80)– 0.63 (0.36, 1.10) and 1.24 (0.63, 2.42)– 1.33 (0.61, 2.89), respectively. **Fig. B**) Density function of ln-transformed ∑As.

The GEE—splines function for the Poisson regression shows an increase of the overall expected counts of respiratory symptoms at high urinary ∑As; however, a greater variability was observed at low concentrations (**Fig 1A**). The density function of ln-transformed urinary ∑As is shown in **Fig 1B**. The crude and adjusted estimates (95% CI) at ln-transformed urinary ∑As 1.57 (average concentration) and 4.00 (99^th^ percentile concentration) were 0.62 (0.48, 0.80)– 0.63 (0.36, 1.10) and 1.24 (0.63, 2.42)– 1.33 (0.61, 2.89) counts, respectively. The increased trend of the overall expected counts at high urinary ∑As was also clear when directly applying Poisson regression at 4 and 7 years of age individually and when modelling the cumulative expected counts of events at both time points **S2 Fig- Poisson regression spline functions between ln-transformed urinary arsenic concentrations (∑As) at 4 years and respiratory symptoms assessed at 4, 7, and 4–7 years of age**. **S3 Fig—Generalized Estimating Equation (GEE)—Poisson regression spline function between natural ln-transformed urinary arsenic concentrations (1. iAs + MMA and 2. DMA) and expected respiratory symptoms at 4 and 7 years of age** gathers the results from the GEE—Poisson regression spline functions when modelling the ln-transformed concentrations of iAs + MMA and DMA separately and shows that urinary DMA drove the increased trend of the overall expected counts.

Increased odds ratios (OR) were also suggested from the association between urinary ∑As and each respiratory symptom of interest when analysed individually and according to the assessment time point (**S4 Fig—Logistic regression spline functions between natural ln-transformed urinary arsenic concentrations (∑As) at 4 years and each respiratory outcome assessed at 4 years of age, S5 Fig—Logistic regression spline functions between natural ln-transformed urinary arsenic concentrations (∑As) at 4 years and each respiratory symptom assessed at 7 years of age, S6 Fig—Logistic regression spline functions between natural ln-transformed urinary arsenic concentrations (∑As) at 4 years and each respiratory symptom assessed at 4 and 7 years of age**, and **S7 Fig—Generalized Estimating Equation (GEE)–logistic regression spline function between natural ln-transformed urinary arsenic (∑As) at 4 years and each respiratory symptom assessed at 4 and 7 years of age**). This was particularly evident for wheeze, wheeze medication, eczema, and sneeze.

## 4. Discussion

This exploratory study found that even at relatively low iAs exposure during childhood, evaluated with urinary ∑As concentrations from 4-year-old children living in Spain, was associated with an increased trend of self-reported respiratory-related outcomes including asthma, eczema, sneeze, and wheeze from follow-up assessments at 4 and 7 years of age.

Here, the study children’s median urinary ∑As was ~10-fold lower than that reported in 4.5- and 9-years old children from the MINIMat cohort in rural Bangladesh exposed to arsenic-contaminated drinking water [39]. Contrary, the urinary concentrations were similar to other child populations consuming water that complies with the EU, US, and WHO maximum arsenic level of <10 μg/L [11–13, 60]. The MINIMat study evaluated prenatal arsenic exposure and reported that *in utero* exposure related to impaired lung function, while childhood exposure increased airway inflammation [39]. In the US, gestational low-level arsenic exposure was associated with an increased risk of respiratory infections involving medical treatment, and respiratory symptoms over the first year of life, and lung function in approximately 7-year-old children [9, 29]. A doubling of maternal urinary ∑As was associated with an estimate of −0.08 decrease in forced vital capacity (FVC) z-scores and −0.10 forced expiratory volume in the first second of exhalation (FEV1) z-scores in a study population of 358 children [29].

Early-life exposure to iAs while lung formation is particularly critical and may adversely impact lifelong respiratory health [61–64]. However, the exact mechanisms of iAs-induced respiratory symptoms and lung function impairment are not fully understood [20]. Inorganic arsenic accumulates in the lungs, kidney, and liver [65–67] and could cause damage in lung tissue by inducing inflammation [3, 39, 68], and generate oxidative stress [69–71]. IgE production is a marker of allergic response generated by Th2 cells, which have been related to increased urinary arsenic concentrations [72, 73]. Increased urinary arsenic concentrations have also been associated with reduced percentages of CD4 T cells and interleukin (IL)-2 secretion levels [74] and T-cell proliferation and cytokine secretion that could cause immunosuppression [75]. The respiratory health effects related to chronic low-level iAs exposure such as that in the Spanish population and others still need to be assessed and characterised. This is among the first studies that prospectively explore low-level iAs exposure during childhood and respiratory outcomes including asthma, asthma medication, wheeze, wheeze medication, sneeze, and eczema.

The children included in this study had access to low arsenic drinking water [11, 42], and thus food intake was likely the major source of iAs exposure [15]. Positive associations between rice consumption and urinary iAs and MMA, and between fish/seafood intake and urinary AsB concentrations were previously reported in the INMA children [42]. In a US cohort, each month earlier of introduction of rice cereals during infants’ first year of live was associated with increased risk of subsequent upper respiratory tract infections, lower respiratory tract infections, acute respiratory symptoms including wheeze, difficulty breathing, and cough, fever requiring a prescription medicine and allergy diagnosed by a physician [44]. Contrary, a diet rich in antioxidants and polyunsaturated fatty acids from consumption of fish, fruits, vegetables, legumes, nuts, and cereals may reduce symptoms of asthma or allergic rhinitis in children [76]. The findings here suggest an increased number of respiratory symptoms in relation to elevated iAs exposure levels after adjustment for consumption of vegetables, fruits, and fish and other covariates. However, the dual role of early life diet in iAs exposure burden and prevention of adverse respiratory outcomes requires further research.

The findings of this study derive from a well-established birth cohort [50, 51] and show a positive trend between the overall counts of respiratory symptoms of interest assessed at 4 and 7 years of age and children’s urinary ∑As concentrations, especially among participants with higher exposure levels. Exposure to iAs and respiratory symptoms were assessed together at 4 years of age. Thus, reverse causality at 4 years cannot be ruled out, however it is not expected to alter the main findings of the study. The modest study size here precluded the analysis of certain specific respiratory outcomes of interest (as shown in the Support Information) and may also have weaken our statistical power to detect associations. This study used one-time spot urine sample arsenic species concentrations adjusted for urine dilution as a proxy for iAs internal exposure. Yet, among populations with consistent patterns of exposure, single samples urinary arsenic concentrations show temporal stability as a result of chronic exposure [77, 78]. A poor agreement (i.e, low ICC) was observed among the arsenic components of ∑As, which was dominated by DMA suggesting that for our low-level exposed population most of the internal iAs was excreted as DMA. The higher proportion of urinary DMA compared to iAs, and MMA in ∑As is in line with prior studies [43, 79]. It was not possible to differentiate urinary DMA from iAs methylation and that from other sources such as direct dietary ingestion or from the metabolism of other organosenical compounds (e.g., arsenosugars and arsenolipids) and that must be taken into account in the interpretation of the results [35, 80]. However, there is a growing body of evidence suggesting that even direct exposure to DMA or from the metabolism of organosenical compounds could exhibit toxic effects similar to iAs [1, 15, 35, 81–83]. Lastly, children’s respiratory symptoms information was gathered through face-to-face interviews by trained study staff; however, it is still prone to parental recall bias.

The findings of this exploratory study highlight the potential detrimental impact of early life relatively low iAs exposure on respiratory health. However, the study has limitations including a modest sample size that might limit the statistical power, and thus some caution must be taken in the interpretation of the findings. Large prospective studies on the effects of chronic low-level iAs exposure are warranted.

## Supporting information

S1 Fig Directed acyclic graph or causal Bayesian network created using DAGitty browser-based environment. (DOCX)

S2 Fig Poisson regression spline functions between ln-transformed urinary arsenic concentrations (∑As) at 4 years and respiratory symptoms assessed at 4, 7, and 4–7 years of age. (DOCX)

S3 Fig Generalized Estimating Equation (GEE)—Poisson regression spline function between natural ln-transformed urinary arsenic concentrations (1. iAs + MMA and 2. DMA) and expected respiratory symptoms at 4 and 7 years of age. (DOCX)

S4 Fig Logistic regression spline functions between natural ln-transformed urinary arsenic concentrations (∑As) at 4 years and each respiratory outcome assessed at 4 years of age. (DOCX)

S5 Fig Logistic regression spline functions between natural ln-transformed urinary arsenic concentrations (∑As) at 4 years and each respiratory symptom assessed at 7 years of age. (DOCX)

S6 Fig Logistic regression spline functions between natural ln-transformed urinary arsenic concentrations (∑As) at 4 years and each respiratory symptom assessed at 4 and 7 years of age. (DOCX)

S7 Fig Generalized Estimating Equation (GEE)–logistic regression spline function between natural ln-transformed urinary arsenic (∑As) at 4 years and each respiratory symptom assessed at 4 and 7 years of age. (DOCX)
