# Supplementary material for: Arsenic exposure and respiratory outcomes during childhood in the INMA study
Source: PLoS One. 2022 Sep 9;17(9):e0274215. doi: 10.1371/journal.pone.0274215 (PMC9462567; doi:10.1371/journal.pone.0274215)
Supplement: S1 Fig — (DOCX) [file pone.0274215.s001.docx]

## Fig S1: Directed acyclic graph or causal Bayesian network created using DAGitty browser-based environment.


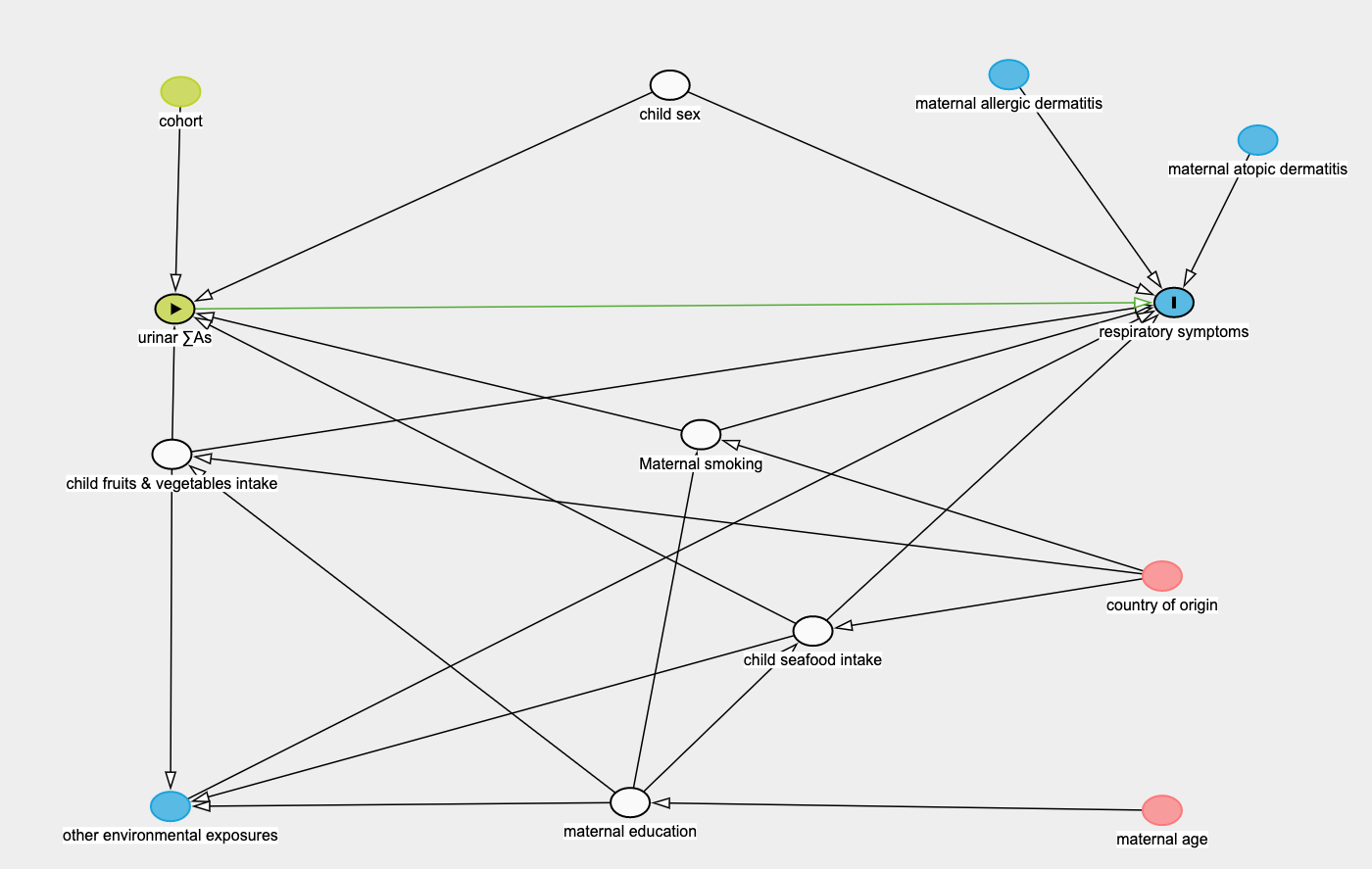


Green circle with ▶︎ = exposure; blue circle with **I** = outcome; green circle = ancestor of exposure; blue circle = ancestor of outcome; red circle = ancestor of exposure and outcome; white circle = adjusted variable; Green arrow = casual pathway.
