# Supplementary material for: Arsenic exposure and respiratory outcomes during childhood in the INMA study
Source: PLoS One. 2022 Sep 9;17(9):e0274215. doi: 10.1371/journal.pone.0274215 (PMC9462567; doi:10.1371/journal.pone.0274215)
Supplement: S2 Fig — (DOCX) [file pone.0274215.s002.docx]

## Fig S2: Poisson regression spline functions between ln-transformed urinary arsenic concentrations (∑As) at 4 years and respiratory symptoms assessed at 4, 7, and 4-7 years of age.


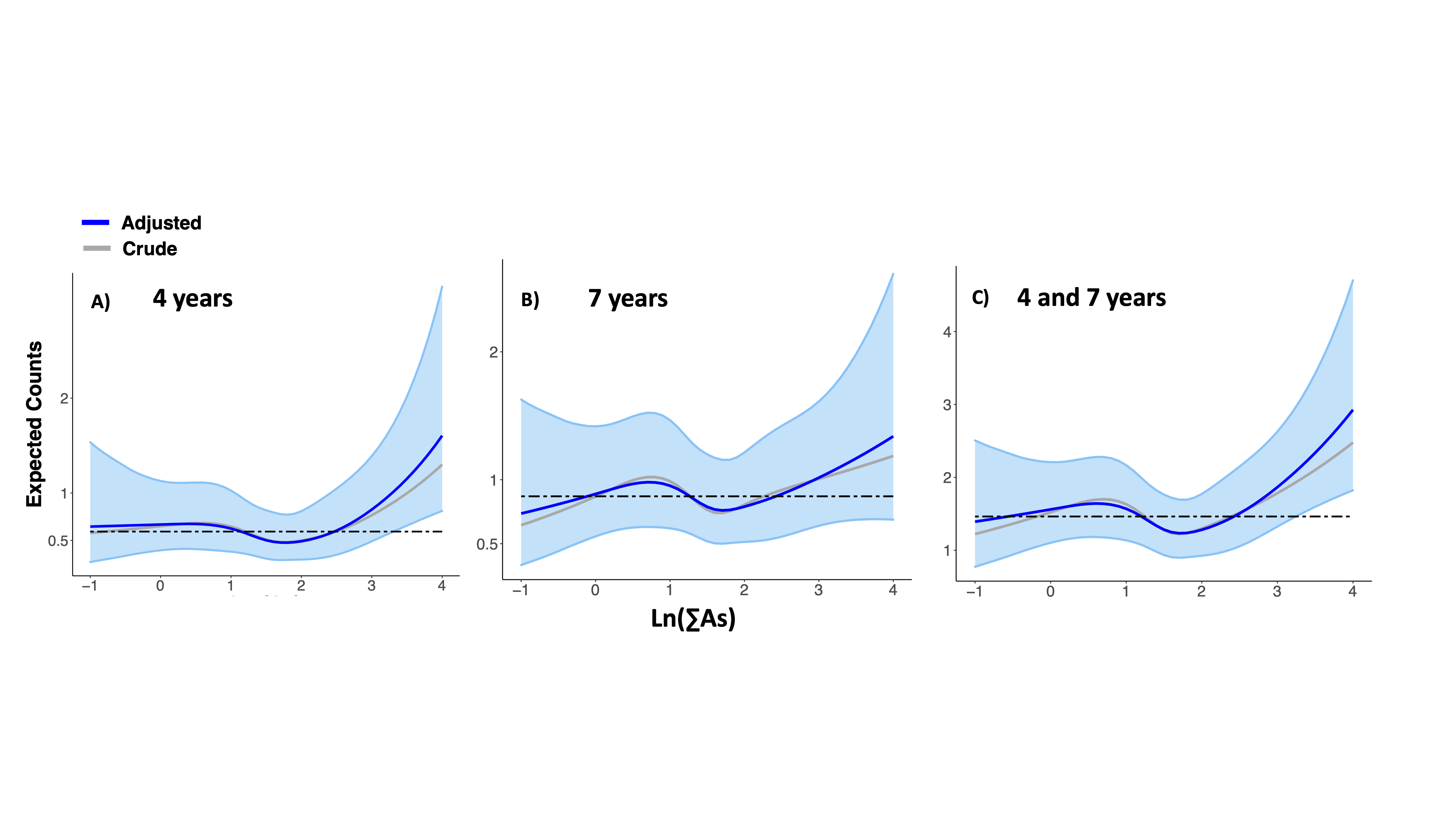


The ∑As is in µg/L. Case-complete approach (i.e., participants with missing values in the dependent, independent, or adjustment variables not included in the analysis). ∑As = iAs + MMA + DMA adjusted for specific gravity. **Fig.** **A**) At 4 years, 216 individuals out of 339 (63.7%) did not have any of the outcomes of interest, while 73 (21.6%) had one, and 50 (14.7%) had ≥2 outcomes. The black dashed line shows the average expected counts (0.59). To facilitate interpretation, the crude and adjusted estimates and 95% confidence intervals at ln-transformed urinary ∑As 1.57 (average concentration), and 4.00 (99^th^ percentile concentration) were 0.50 (0.39, 0.65) – 0.55 (0.30, 0.80) and 1.30 (0.75, 2.25) – 1.60 (0.81, 3.17), respectively. **Fig. B**) At 7 years, 170 out of 339 (50.1%) did not have any of the outcomes of interest, while 101 (29.8%) had one, and 68 (20.1%) had ≥2 outcomes. The black dashed line shows the average expected counts (0.87). To facilitate interpretation, the crude and adjusted estimates (95% confidence intervals) at ln-transformed urinary ∑As 1.57 (average concentration), and 4.00 (99^th^ percentile concentration) were 0.75 (0.60, 0.94) – 0.78 (0.51, 1.18) and 1.19 (0.68, 2.08) – 1.34 (0.69, 2.61), respectively. **Fig. C**) In the compiled analysis counting respiratory symptoms at 4 and 7 years, 134 out of 339 (39.6%) did not have any of the outcomes of interest, while 91 (26.8%) had one, and 114 (33.6 %) had ≥2 outcomes. The black dashed line shows the average expected counts (1.46). To facilitate interpretation, the crude and adjusted estimates (95% confidence intervals) at ln-transformed urinary ∑As 1.57 (average concentration) and 4.00 (99^th^ percentile concentration) were 1.25 (1.05, 1.48) – 1.33 (0.91, 1.73) and 2.48 (1.67, 3.67) – 2.93 (1.82, 4.70), respectively. The grey lines show the crude models. The dark blue lines show the adjusted models for child sex (boys or girls), and maternal smoking status (“Have you ever smoke?” - binary) and level of education (primary, secondary, or university studies), and cohort (Asturias, Gipuzkoa, Sabadell, or Valencia), and calorie adjusted consumption of vegetables (g/day), fruits (g/day) and fish/seafood (g/day) at 4 (**Fig. A**), 7 (**Fig. B**), or 4 and 7 years of age (**Fig. C**). The blue shades show the 95% confidence interval. The black dashed lines show the average expected counts. Notice that the scale of y-axis varies to facilitate the visualization of the expected counts in each plot.
