# Supplementary material for: Arsenic exposure and respiratory outcomes during childhood in the INMA study
Source: PLoS One. 2022 Sep 9;17(9):e0274215. doi: 10.1371/journal.pone.0274215 (PMC9462567; doi:10.1371/journal.pone.0274215)
Supplement: S3 Fig — (DOCX) [file pone.0274215.s003.docx]

## Fig S3: Generalized Estimating Equation (GEE) - Poisson regression spline function between natural ln-transformed urinary arsenic concentrations (1. iAs + MMA and 2. DMA) and expected respiratory symptoms at 4 and 7 years of age.


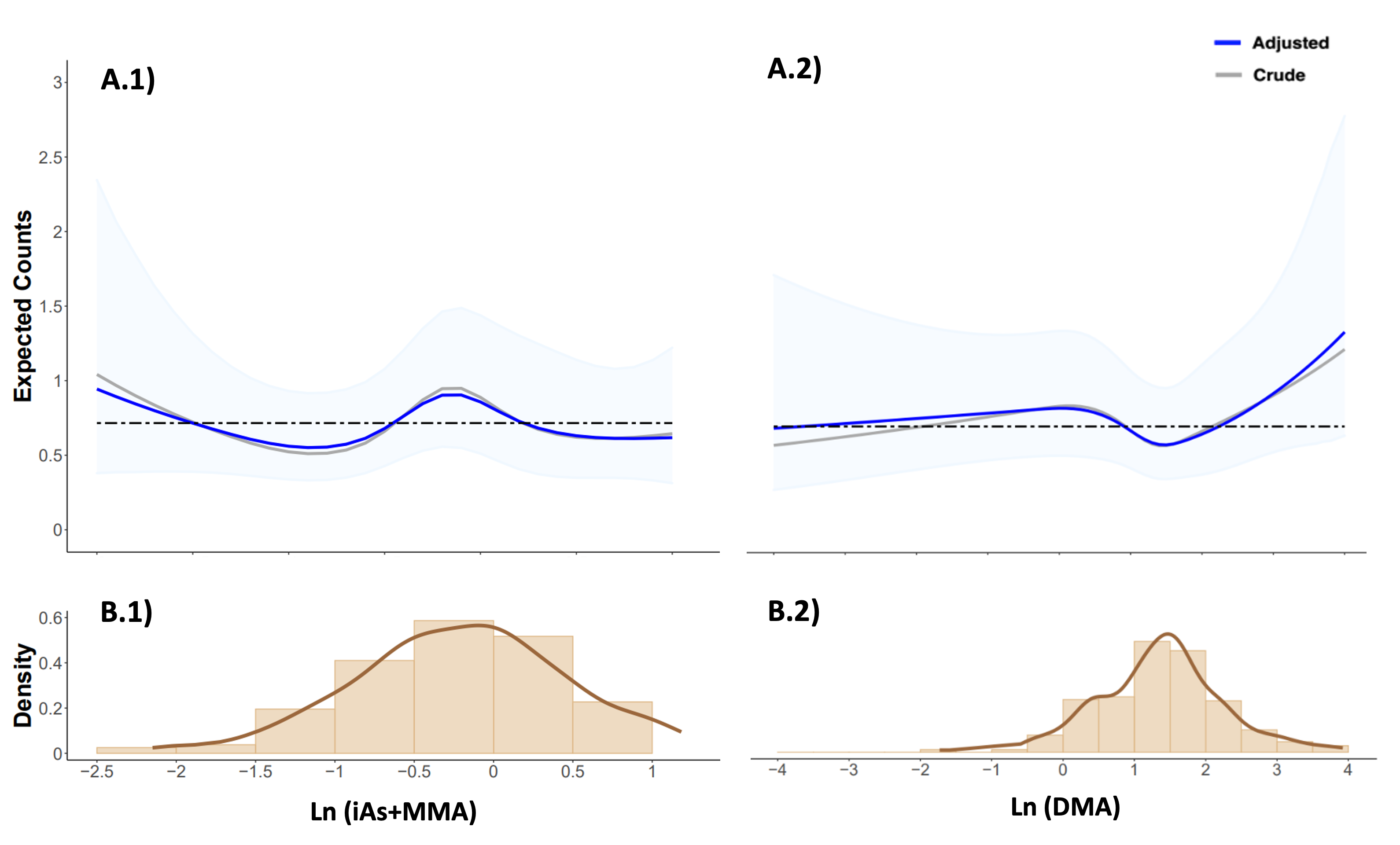


The iAs + MMA and DMA are in µg/L. Case-complete approach (i.e., participants with missing values in the dependent, independent, or adjustment variables were not included in the analysis) of *n* = 339. The concentrations of iAs, MMA, DMA were adjusted for specific gravity. **Fig. A.1** and **Fig. A.2** show the Generalized Estimating Equation (GEE) using family equal Poisson and splines functions. Respiratory symptoms include asthma, asthma medication, wheeze, wheeze medication, sneeze, and eczema. The grey line shows the crude model. The dark blue line shows the adjusted models for child sex (boys or girls), and maternal smoking status (“Have you ever smoked?” - binary) and level of education (primary, secondary, or university studies), cohort (Asturias, Gipuzkoa, Sabadell, or Valencia), and calorie adjusted consumption of vegetables (g/day), fruits (g/day) and fish/seafood (g/day) at 4 and 7 years of age. The blue shade shows the 95% confidence interval of the adjusted model. The black dashed line shows the average expected counts. **Fig. B.1** and **Fig. B.2** show the density function of ln-transformed iAs + MMA and DMA, respectively.
