# Supplementary material for: Arsenic exposure and respiratory outcomes during childhood in the INMA study
Source: PLoS One. 2022 Sep 9;17(9):e0274215. doi: 10.1371/journal.pone.0274215 (PMC9462567; doi:10.1371/journal.pone.0274215)
Supplement: S4 Fig — (DOCX) [file pone.0274215.s004.docx]

## Fig S4: Logistic regression spline functions between natural ln-transformed urinary arsenic concentrations (∑As) at 4 years and each respiratory outcome assessed at 4 years of age.


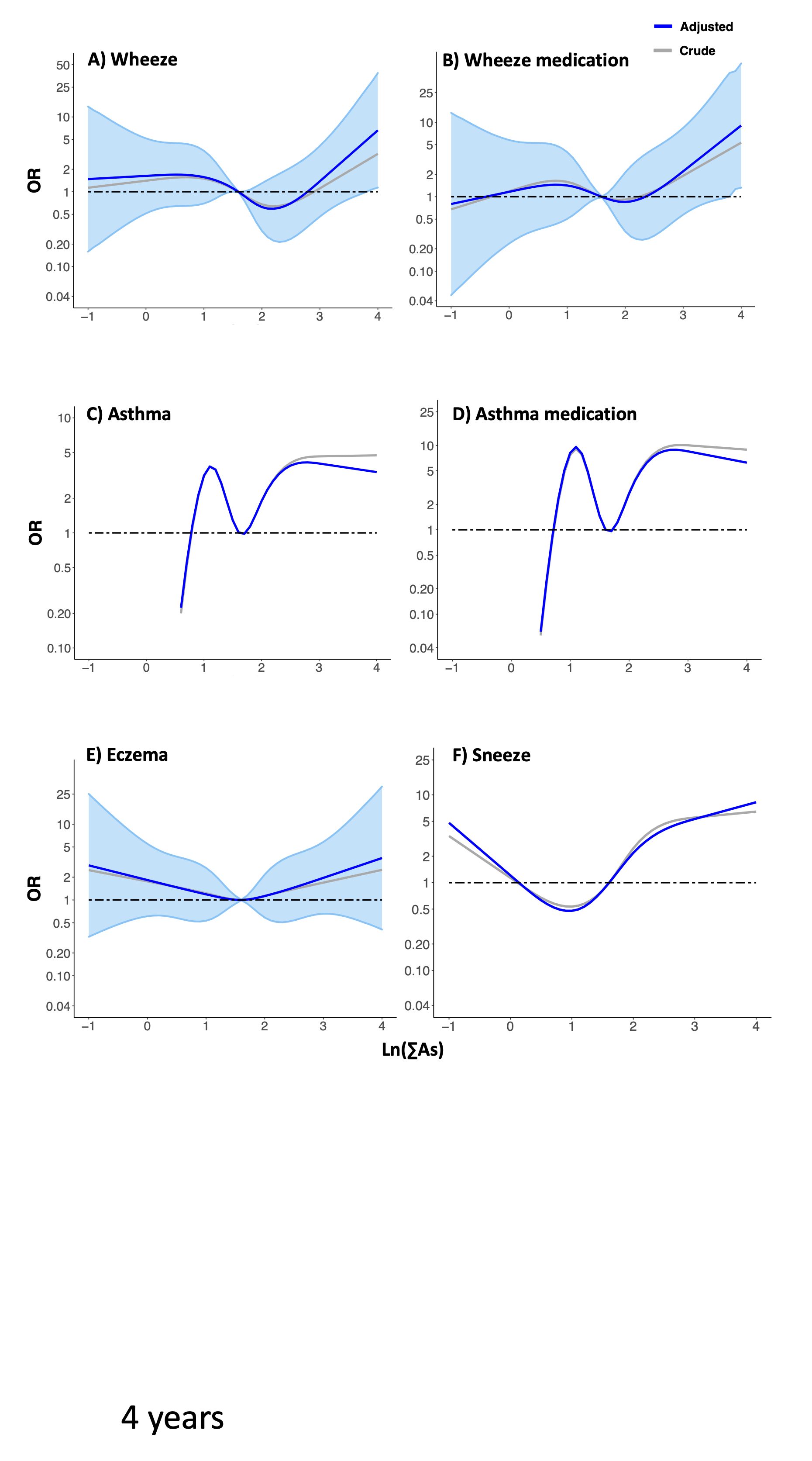


The ∑As is in µg/L. Case-complete approach (i.e., participants with missing values in the dependent, independent, or adjustment variables not included in the analysis). The median ∑As was used as the reference value. The grey lines show the crude models. The dark blue lines show the adjusted models for child sex (boys or girls), and maternal smoking status (“Have you ever smoke?” - binary) and level of education (primary, secondary, or university studies), cohort (Asturias, Gipuzkoa, Sabadell, or Valencia), and calorie adjusted consumption of vegetables (g/day), fruits (g/day) and fish/seafood (g/day) at 4 years of age. The blue shades show the 95% confidence interval. The black dashed lines show the null. Logistic regression model between ln-transformed summation of urinary arsenic species (∑As = iAs + MMA + DMA) adjusted for specific gravity at 4 years and wheeze (binary) (**Fig. A**), wheeze (binary) (**Fig. B**), asthma (binary) (**Fig. C**), asthma medication (binary) (**Fig. D**), eczema (binary) (**Fig. E**), and sneeze (binary) (**Fig. F**) assessed at 4 years of age. The confidence intervals are not included when they are not informative due to the low number of cases (e.g., 9 cases of asthma medication out of 334, and 10 cases of asthma out of 337, and 7 cases of sneeze out of 334). Notice that the scale of the y-axis varies to facilitate the visualization of the estimates in each plot.
