# Supplementary material for: Arsenic exposure and respiratory outcomes during childhood in the INMA study
Source: PLoS One. 2022 Sep 9;17(9):e0274215. doi: 10.1371/journal.pone.0274215 (PMC9462567; doi:10.1371/journal.pone.0274215)
Supplement: S5 Fig — (DOCX) [file pone.0274215.s005.docx]

## Fig S5: Logistic regression spline functions between natural ln-transformed urinary arsenic concentrations (∑As) at 4 years and each respiratory symptom assessed at 7 years of age.


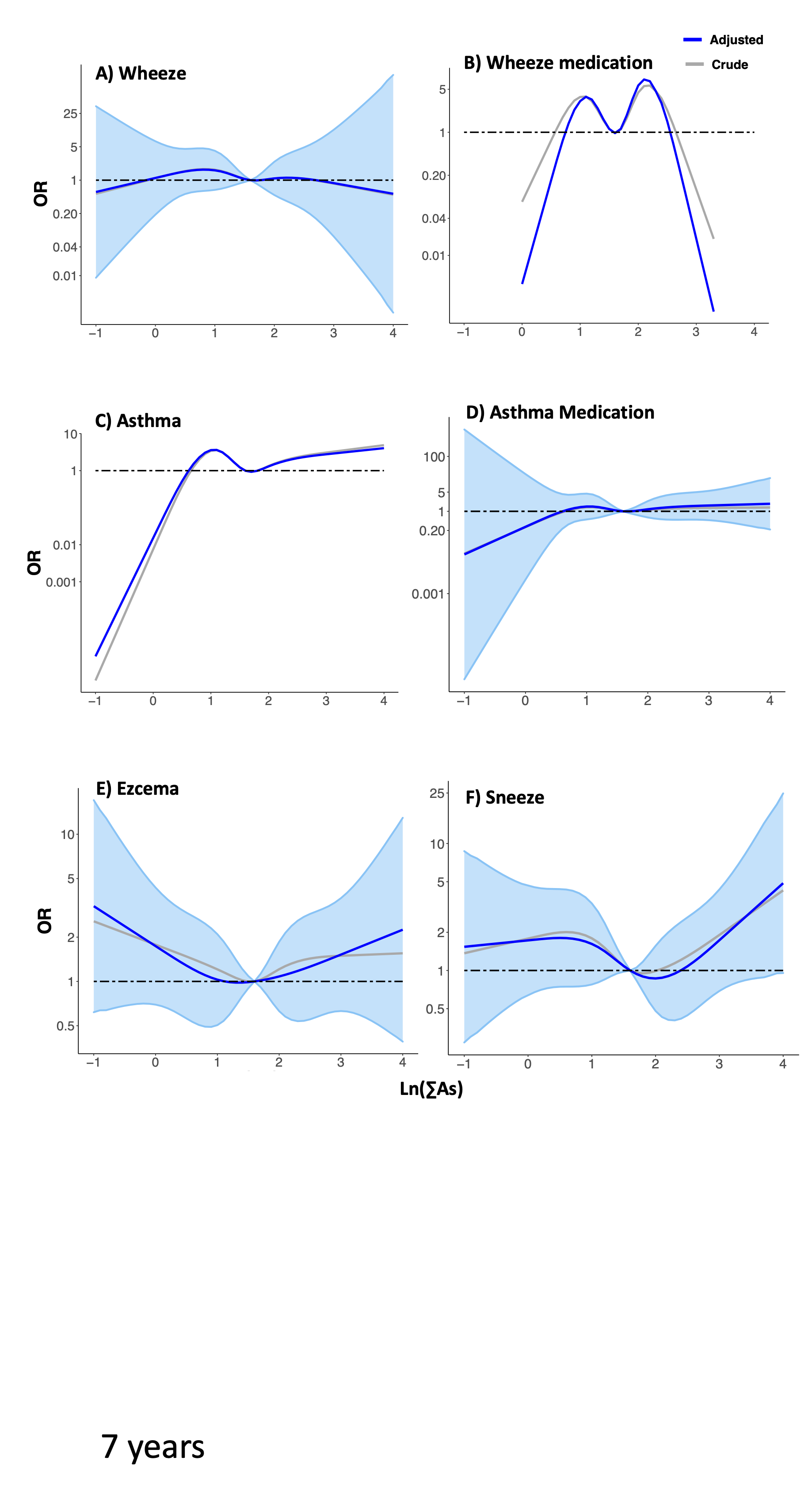


The ∑As is in µg/L. Case-complete approach (i.e., participants with missing values in the dependent, independent, or adjustment variables were not included in the analysis). The median ∑As was used as the reference value. The grey lines show the crude models. The dark blue lines show the adjusted models for child sex (boys or girls), and maternal smoking status (“Have you ever smoke?” - binary) and level of education (primary, secondary, or university studies), cohort (Asturias, Gipuzkoa, Sabadell, or Valencia), and calorie adjusted consumption of vegetables (g/day), fruits (g/day) and fish/seafood (g/day) at 7 years of age. The grey lines show the crude models. The dark blue lines show the adjusted models for child sex (boys or girls), and maternal smoking status (“Have you ever smoke?” - binary) and level of education (primary, secondary, or university studies). The blue shades show the 95% confidence interval. The black dashed lines show the null. Logistic regression model between ln-transformed summation of urinary arsenic species (∑As = iAs + MMA + DMA) adjusted for specific gravity at 4 years and wheeze (binary) (**Fig A**), wheeze medication (binary) (**Fig B**), asthma (binary) (**Fig C**), asthma medication (binary) (**Fig D**), eczema (binary) (**Fig F)**, and sneeze (binary) (**Fig F)** assessed at 7 years of age. The confidence intervals are not included when they are not informative due to the low number of cases (e.g., 5 cases of wheeze medication out of 93 and 18 cases of asthma out of 337). Also, the assessment of wheeze medication was not available in all cohorts and thus the model for wheeze is not adjusted for cohort. Notice that the scale of the y-axis varies to facilitate the visualization of the estimates in each plot.
